# Theories and Molecular Basis of Vascular Aging: A Review of the Literature from VascAgeNet Group on Pathophysiological Mechanisms of Vascular Aging

**DOI:** 10.3390/ijms23158672

**Published:** 2022-08-04

**Authors:** Eugenia Gkaliagkousi, Antonios Lazaridis, Soner Dogan, Emil Fraenkel, Bilge Guvenc Tuna, Ioana Mozos, Milica Vukicevic, Ozlem Yalcin, Kristina Gopcevic

**Affiliations:** 1 3rd Department of Internal Medicine, Papageorgiou Hospital, Faculty of Medicine, Aristotle University of Thessaloniki, 56429 Thessaloniki, Greece; 2 Department of Medical Biology, School of Medicine, Yeditepe University, 34755 Istanbul, Turkey; 3 1st Department of Internal Medicine, University Hospital, Pavol Jozef Šafárik University of Košice, Trieda SNP 1, 04066 Košice, Slovakia; 4 Department of Biophysics, School of Medicine, Yeditepe University, 34755 Istanbul, Turkey; 5 Department of Functional Sciences-Pathophysiology, Center for Translational Research and Systems Medicine, “Victor Babes” University of Medicine and Pharmacy, 300173 Timisoara, Romania; 6 Cardiac Surgery Clinic, Clinical Center of Serbia, 11000 Belgrade, Serbia; 7 Department of Physiology, School of Medicine, Koc University, 34450 Istanbul, Turkey; 8 Laboratory for Analytics of Biomolecules, Department of Chemistry in Medicine, Faculty of Medicine, 11000 Belgrade, Serbia

**Keywords:** vascular aging, inflammation, atherosclerosis, matrix metalloproteinases, oxidative stress

## Abstract

Vascular aging, characterized by structural and functional alterations of the vascular wall, is a hallmark of aging and is tightly related to the development of cardiovascular mortality and age-associated vascular pathologies. Over the last years, extensive and ongoing research has highlighted several sophisticated molecular mechanisms that are involved in the pathophysiology of vascular aging. A more thorough understanding of these mechanisms could help to provide a new insight into the complex biology of this non-reversible vascular process and direct future interventions to improve longevity. In this review, we discuss the role of the most important molecular pathways involved in vascular ageing including oxidative stress, vascular inflammation, extracellular matrix metalloproteinases activity, epigenetic regulation, telomere shortening, senescence and autophagy.

## 1. Introduction

Aging is a natural physiological process characterized by the progressive loss of tissue and organ function [1]. The aging rate around the world is increasing dramatically and is accompanied by an increase in mortality due to main age-associated diseases [2]. More importantly, aging represents the main risk factor for cardiovascular disease (CVD) which carries the highest burden for the older population and is the leading cause of death worldwide [3,4]. In addition, the gradual decrease in the adaptive abilities of the organism, which is a basic manifestation of aging, can play a significant role in the development of several other pathologies including malignant diseases, neurodegenerative processes, reduced resistance to infection and diabetes mellitus [5]. 

In particular, vascular aging is a gradually developing process characterized by alterations in the properties of the vascular wall that start very early in life. In fact, it has been documented that the architecture of the vascular system is programmed in utero and most of the elastin, the major structural component underlying arterial wall elasticity, is synthesized, and deposited during that period. At the same time, it has been demonstrated that disorganization of elastic fibers and therefore alterations in vascular structure as well as hemodynamic function, appear early in human fetal aorta and continue during postnatal life, being extended immediately after birth [6,7]. In accordance with this, marked impairments in the vascular structure and function have been described in children and adolescents with low birth weight as well as in cases of prematurity and intrauterine growth retardation resulting in a small for gestational age phenotype [8,9].

Finally, the phenotype of vascular aging in adults will be identified by certain vascular alterations which result in vascular dysfunction and development of a wide range of age-related vascular pathologies. These alterations are divided into structural changes which include the progressive thickening of the vascular wall along with vascular smooth muscle cell (VSMC) migration and proliferation, namely vascular remodeling, and the functional changes which include endothelial dysfunction, loss of arterial elasticity and reduced arterial compliance, all of which result in increased arterial stiffness [10,11]. 

The pathogenesis behind these changes in vascular aging involves multiple complex cellular and molecular mechanisms such as mitochondrial dysfunction and oxidative stress, inflammation, loss of proteostasis, genomic instability, cellular senescence, increased apoptosis and necroptosis, epigenetic alterations, and extracellular matrix (ECM) remodeling [12,13].

As many age-related cardiovascular and cerebrovascular diseases are due to alterations in vascular function or are exacerbated by vascular functional and structural changes, it is important to thoroughly elucidate those fundamental pathophysiological mechanisms underlying the vascular aging process, in an attempt to develop novel treatments to reduce age-associated mortality. In this review, we describe the fundamental cellular and molecular mechanisms of aging: oxidative stress, chronic low-grade inflammation, cell matrix injury, epigenetic alterations, telomere length, cellular senescence and autophagy, considering in vitro and in vivo preclinical research and clinical studies.

## 2. Methodology

In an attempt to identify all relevant studies, we conducted a thorough search of the literature using PubMed. The search strategy used mainly the terms “pathophysiolocigal mechanisms”, “molecular mechanisms”, and “vascular aging,” and the initial selection was refined by the major mechanisms extensively described in the literature. In addition, we opted to include those mechanisms demonstrating a causal relationship with the essential alterations in the properties of the vascular wall that define vascular aging, including endothelial dysfunction, atherosclerosis and vascular stiffness. Research articles were selected manually from the reference lists of relevant articles. The abstracts and titles of the articles retrieved were screened to exclude the irrelevant studies. 

### 2.1. Oxidative Stress

#### 2.1.1. Role of Oxidative and Nitrosative Stress 

**Figure 1:**
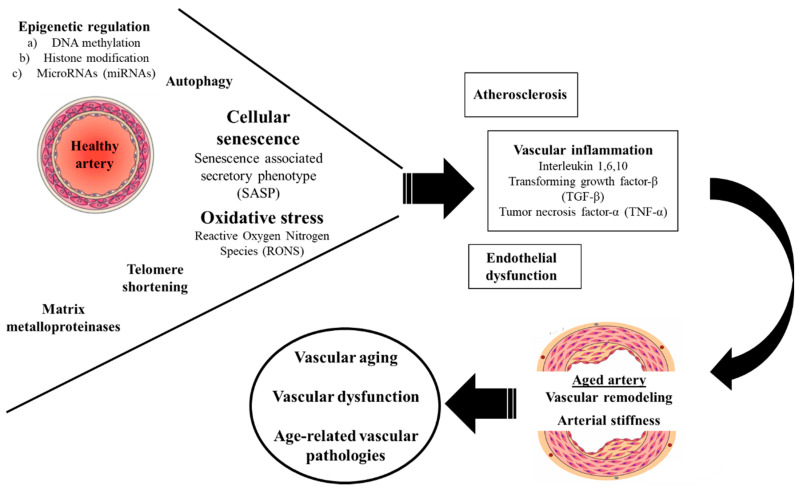
Molecular mechanisms of vascular aging. Oxidative stress, cellular senescence, telomere shortening, epigenetic regulation, matrix metalloproteinases and autophagy represent the main pathophysiological mechanisms mediating inflammation, atherosclerosis and endothelial dysfunction, finally leading to vascular ageing.

The main source of free radicals is oxygen. Free radicals, characterized by the loss of one electron in the molecules, are continuously formed as a consequence of numerous oxidative chemical reactions. Oxidative stress, which is a consequence of imbalance between production and detoxification of reactive oxygen and nitrogen species (RONS), is one of the underlying factors in several diseases as well as one of the hallmarks of aging [14,15] (Figure 1).

Normally, in a healthy organism, homeostatic RONS concentrations play a crucial role as secondary messengers in many intracellular signaling pathways in both innate and adaptive immune responses [14]. Under conditions of increased RONS concentration, mainly produced as a consequence of mitochondrial dysfunction, detoxifiers are not able to completely remove them, leading to cellular damage, tissue injury, and inflammation. Thus, oxidative stress has been associated with the pathogenesis of endothelial dysfunction, atherosclerosis and several chronic diseases [16,17]. The spectrum of oxygen reactive species that are considered responsible for biological oxygen toxicity include the intermediates of the partial reduction of oxygen, superoxide radical (O_2_^•−^), hydrogen peroxide (H_2_O_2_), and other reactive species as hydroxyl radicals (HO^•^), peroxyl radical (ROO^•^), nitric oxide (NO), peroxinitrite (ONOO^−^) and singlet oxygen (^1^O_2_) [18]. In Table S1 (Supplementary) are presented RONS species, radicals and nonradicals, their formation, characteristics and detoxification.

#### 2.1.2. Endogenous Sources of RONS 

The endogenous sources of RONS include the nicotinamide adenine dinucleotide phosphate (NADPH) oxidase, myeloperoxidases (MPOs), lipoxygenases (LPOs) and angiotensin II (AngII) [19]. They are produced by various organelles within the cell in response to several physiological stimuli (i.e., exercise) or pathophysiological conditions including inflammation, degenerative and age-associated diseases. NADPH oxidases are the main source of the superoxide anion which is formed by the one-electron reduction of molecular oxygen. Superoxide dismutase (SOD) dismutates the superoxide anion into hydrogen peroxide (H_2_O_2_) which is able to form highly reactive hydroxyl ions (OH^•^), that are extremely reactive and cause damage to the cell membrane phospholipids and proteins [20,21]. MPO is involved in the formation of products derived from the oxidation of arachidonic acid which are involved in the inflammatory response and in lipid peroxidation [22], and all these compounds contribute to oxidative stress by oxidizing low density lipoprotein (LDL) and lowering NO bioavailability [19]. MPO promotes atherogeneis through the production of modified subtypes of LDL and high density lipoprotein (HDL) [23,24,25]. On the other hand, LPOs have a potential role in the pathogenesis of atherosclerosis by peroxidation of polyunsaturated fatty acids into bioactive lipids and several forms of LPOs were found to be overexpressed in atherosclerotic lesions [26]. Finally, another reactive molecule, peroxinitrite (ONOO^−^) can be formed during the reaction of O_2_ with NO, which is produced from L-arginine by the endothelial, neuronal and inducible NO synthases (NOS) [17].

#### 2.1.3. The Renin/Angiotensin Signaling Pathway

The renin/angiotensin system (RAS) is the basic signaling pathway participating in vascular aging [27]. An important source of free radicals is AngII which is a product of AngI cleavage by angiotensin converting enzyme (ACE). Originally described as a potent vasoconstrictor, AngII is now recognized as a multifunctional hormone influencing many cellular processes which are crucial for the regulation of vascular function, including cell growth, apoptosis, migration, inflammation, and fibrosis [28,29]. The expression and activity of the angiotensin-converting enzyme-1 (ACE-1) significantly increases in both endothelial cells (ECs) and VSMCs during aging. Current evidence suggests that Ang II, through Ang II type 1 (AT1)-receptor activation, can stimulate intracellular formation of reactive oxygen species (ROS) such as the superoxide anion (O_2_^•−^) by involving membrane-bound NAD(P)H-oxidases, hence resulting in an increase in intracellular O_2_^•−^ concentration [30]. AT1 levels increase within the aged arterial wall. In contrast, the expression of ACE2 decreases with age, thus reducing its inhibitory effect on the RAS, an effect which might lead to higher expression levels of AngII and significant Ang II-related vascular alterations [31]. Thus, the overall activity of the RAS is increased in the elderly. 

#### 2.1.4. Exogenous Sources of RONS

Apart from the endogenous production, exogenous sources of RONS are: heavy or transition metals, radiation, drugs, air and water pollution [21]. Biological systems are protected from RONS toxicity by endogenous enzymes such as SOD, catalase (CAT) and glutathione-peroxidase (GPX) molecules which represent the first line of defense against free radicals, but also nonenzymatic (i.e., vitamin E, bilirubin, β-carotene, albumin) and exogenous molecules (vitamins C and E, phenolic antioxidants, selenium, zinc) [32]. 

### 2.2. Inflammation

Chronic low-grade inflammation is considered as one of the main mechanisms underlying biology of vascular aging through multiple mechanisms including endothelial dysfunction, atherosclerosis, increased vascular stiffness and vascular calcification [33]. Accordingly, inflammaging is a hypothesis suggesting a link between increased pro-inflammatory marker levels and increased risk for cardiovascular disease in older age [34]. Indeed, increased levels of pro-inflammatory serum markers in the circulation of older individuals including interleukins (IL, -1, -6, -8, -13, -18), chemokines (RANTES, macrophage inflammatory protein-1 alpha [MIP-1a], monocyte chemotactic protein-1 [MCP-1]), C-reactive protein (CRP), interferon alpha and beta (IFN-α, IFN-β), transforming growth factor-β (TGF-β), and tumor necrosis factor (TNF), have been found to be associated with vascular aging [35,36,37] and, subsequently, with indices of vascular dysfunction [38,39,40]. Below, the major inflammatory molecules as well as the anti-inflammatory molecule IL-10, all associated with vascular aging, are discussed. 

#### 2.2.1. Interleukin-1

Interleukin-1 (IL-1), including IL-1α and IL-1β, is a multifunctional cytokine that is consistently induced following tissue injury and inflammation. In this regard, IL-1 activation through the nuclear factor-κB (NF-κB) system, mediates the transcription and production of ample inflammatory factors, including pro-inflammatory cytokines, chemokines, and adhesion molecules, therefore representing a significant mediator of the inflammatory response. The major IL-1 producing cells are macrophages, however, other cells like neutrophils, ECs, and fibroblasts are able to synthesize IL-1 [41].

So far, levels of IL-1 have not been found to elevate with aging. Instead, increased levels of IL-receptor antagonist (IL-1ra) have been observed in older individuals [42]. However, IL-1 certainly exerts a significant inflammatory impact on vascular hemostasis which primarily relies on its pro-atherogenic effect and its ability to regulate some key inflammatory factors including leukocyte chemotaxis and adhesion. To this extend, it has been observed in apolipoprotein E (ApoE) deficient mice that deficiency of IL-1ß decreases monocyte infiltration into the subendothelial space and the formation of atherosclerotic lesions, presumably through reduced vascular expression of vascular cell adhesion molecule-1 (VCAM-1) and MCP-1 [43]. In a similar experimental model, deficiency of IL-1ra resulted in significant and uncontrolled vascular inflammation evidenced by prominent macrophage infiltration in the adventitia and severe destruction of the elastic lamina [44]. Comparable results were also drawn by a functional elimination of the IL-1ra gene in mice leading to pronounced arterial inflammation which was corroborated by the presence of intense vascular infiltration from inflammatory cells [45]. Furthermore, the role of IL-1 in promoting vascular inflammation has been supported by studies showing that IL-1 is a potent inducer of vascular permeability either by the loss of cell-cell junction components such as β-catenin and VE-cadherin, specifically at the EC border, or indirectly through induction of the pro-permeability factor R-spondin 3 (RSPO3) [46]. Finally, IL-1 has been recognized as a potent inducer of Interleukin-6 (IL-6) production and neointimal formation [47]. 

#### 2.2.2. Ιnterleukin-6

IL-6 is a pleiotropic pro-inflammatory cytokine that is produced by several vascular cells (including fibroblasts, ECs and VSMCs) and activates innate and adaptative immunity in response to tissue injury or infection. At a molecular level, its inflammatory effect is mediated by the IL-6 trans-signaling pathway which activates Jak2/Stat3 downstream signaling and leads to increased circulation of adhesion molecules, decreased mitophagy, mitochondrial dysfunction and enhanced vascular permeability [48]. 

It has been consistently reported that aging is associated with increased circulatory levels of IL-6, possibly mediated by increased basal production by VSMCs [49]. Subsequently, IL-6 has been associated with several oxidative and inflammatory mechanisms related to vascular dysfunction and vascular aging per se. To this extent, it has been demonstrated that IL-6 can stimulate endothelial expression of adhesion molecules, including VCAM-1, intercellular adhesion molecule-1 (ICAM-1) and E-selectin, thereby promoting immune cell recruitment and infiltration into the vascular wall and propagating early inflammation [50]. In addition, in cultured ECs, IL-6 has been associated with reduced endothelial NOS (eNOS) expression via a STAT3-mediated inhibition of sequences at amino acid residue-1024. Two other mechanisms linking IL-6 with reductions in NO bioavailability include the association of IL-6 with decreased phosphorylation of eNOS at site Ser1177 as well as an increase in expression of caveolin-1, thus leading to diminished eNOS activity [51]. More importantly, it has been demonstrated that IL-6 deficiency protects against AngII-induced endothelial dysfunction and increases in vascular superoxide [52]. Concurrently, IL-6 exerts a significant positive effect on VSMC proliferation and migration, in part, due to an increase in platelet-derived growth factor (PDGF) and, most importantly, due to oxidative stress, specifically superoxide [50]. In fact, NADPH oxidase is an important source of vascular superoxide in response to IL-6. More specifically, it has been observed that AngII-mediated elevation of superoxide increases levels of IL-6 while at the same time IL-6 promotes an increase in Nox2-derived superoxide. In addition to this, IL-6 upregulates angiotensin type 1 receptor expression [53], leading to even greater production of superoxide, the net result being a vicious cycle of increased IL-6 and superoxide that negatively impacts vascular function. Relative to this, it has been shown that IL-6 deficiency hinders the hypertrophic effect observed upon AngII infusion. Similarly, pharmacological inhibition of the IL-6 signaling cascade results in a blunted hypertrophic response of carotid arteries to AngII [54]. Finally, IL-6 has been implicated as a contributing factor in vascular fibrosis corroborated by the observation that the IL-6 trans signaling pathway leads to elevated levels of transforming growth factor, SMAD3 activation and type I collagen production [55].

#### 2.2.3. Ιnterleukin-10

Interleukin 10 is generally considered a potent anti-inflammatory factor suppressing the actions of IL-6, TNF-α, and IL-8 and one of the key cytokines preventing inflammation-mediated tissue damage [56]. It is produced by an array of leukocytic cell types as well as non-immune cells and exerts its effects by binding to its cognate receptor (IL-10R), thus mainly activating the IL-10/JAK1/STAT3 cascade which is an essential negative regulator of inflammation [57]. 

In age-related disease, it has been demonstrated that IL-10 exerts a vasoprotective effect against endothelial dysfunction and atherogenesis. Relevant to this, evidence of vascular aging has been observed in the carotid arteries of mouse models genetically deficient in IL-10 by means of endothelial dysfunction induced by oxidative stress [58]. Moreover, by modulating the RhoA-Rho kinase pathway, IL-10 exerts a direct effect on VSMCs. As such, in IL-10 knockout (-/-) mice, it has been shown that AngII-infusion results in augmented aortic vascular constriction whereas exogenous IL-10 infusion prevents the aforementioned effect, therefore implying the capability of IL-10 to regulate vascular smooth muscle contraction [59]. Similarly, vascular stiffening as evidenced by increased pulse wave velocity has been documented in aged IL-10 knockout mice [60]. Pertinent to the beneficial impact of IL-10 on vascular homeostasis, its atheroprotective effects have been advocated in experimental studies showing that IL-10 expression can prevent carotid neointima formation [61]. In addition, IL-10 can attenuate atherosclerosis through scavenging of extracellular oxidized LDL (oxLDL) by a lectin-like oxidized low-density lipoprotein receptor-1 (LOX-1)-induced mechanism [62] and the promotion of cholesterol efflux by upregulating the active cellular cholesterol exporters, namely the ATP-binding cassette transporters A1 and G1 [63]. Several other atheroprotective mechanisms of IL-10 such as anti-inflammatory and anti-apoptotic pathways, matrix metalloproteinases (MMPs) and tissue factor inhibition and a modulation of macrophage polarization, have been observed as well [64]. Interestingly, both higher and lower IL-10 serum levels have been reported in association with aging [65,66].

#### 2.2.4. Transforming Growth Factor Beta

Transforming growth factor beta (TGF-β) is a pleiotropic cytokine consisting of three different isoforms (TGF-β1, TGF-β2, and TGF-β3) among which TGF-β1 is the most functional. Accumulating evidence have shown that TGF-β1 exerts a versatile impact on several cellular functions including cell growth, proliferation, senescence, and apoptosis [67]. In addition, TGF-β1 regulates certain mechanisms related to vascular aging such as vascular remodeling and fibrosis [68]. Particularly, by mediating the epidermal growth factor receptor (EGFR)/pp60c-src/MEK-ERK and the Rho/ROCK-dependent SMAD2 pathways in VSMCs, TGF-β1 upregulates the expression of several ECM proteins strongly involved in vascular fibrosis such as fibronectin, type I collagen, connective tissue growth factor (CTGF) and plasminogen activator inhibitor-type 1 (PAI-1) [69]. As a result, the fibrotic TGF-β1 signaling has been linked to vascular stiffening through the induction of significant structural alterations of the VSMCs and the vascular wall. Notably, increased activation of TGF-β1 signaling has been found in the aortic wall with aging and during development of hypertension [70] and TGF-β1 has emerged as a potent mediator of the fibrotic effects of AngII, thus confirming a strong interrelationship between them [71]. Contrary to its profibrotic effects, data has shown that TGF-β1 possess significant anti-inflammatory properties and exerts a rather atheroprotective effect in early atherosclerosis, whereas in the later stages seems to act as a proatherogenic factor by increasing ECM production and inducing pathologic vascular remodeling [72].

#### 2.2.5. Tumor Necrosis Factor-α

TNF-α is a major proinflammatory cytokine predominantly produced by the macrophages as well as a broad variety of other cell types. By binding to its specific receptors (TNFR1, TNFR2), TNF activates discrete intracellular pathways including the NF-κB, mitogen-activated protein kinases (MAPKs) and the apoptotic cascade. Through these pathways, TNF signaling has been implicated in several mechanisms contributing to vascular dysfunction and aging [73]. More specifically, compelling evidence has shown that TNF-α reduces NO production and impairs endothelium-dependent vasodilation by downregulating the expression of eNOS and argininosuccinate synthase enzymes [74]. Endothelial dysfunction is further propagated by a NADPH-dependent overproduction of O_2_^•−^ which is directly induced by TNF. Consistent with these observations, it has been demonstrated that administration of recombinant TNF-α in carotid arteries of young animals elicits endothelial dysfunction, oxidative stress, and increased proinflammatory gene expression, effects that closely mimic the aging-induced functional alterations of the vasculature. On the contrary, chronic TNF-α inhibition with etanercept completely abolishes these effects [75]. Notably, increased circulating plasma levels of TNF-α have been found in elderly individuals [76] whereas enhanced TNF-α expression and production has been demonstrated within the vascular wall (carotid and coronary arteries, aortic wall) [77].

### 2.3. Extracellular Matrix Metalloproteinases

The healthy vasculature comprises of the ECs, VSMCs and the ECM, all of which are susceptible to damage or disruption during aging [78]. The ECM is composed of structural proteins such as collagens and elastin that tether VSMCs together, provide structural support, and regulate the mechanical function of the vessel [79]. Disruption of ECM integrity by MMPs greatly changes its composition and substantially impacts vascular homeostasis during aging through structural and functional changes of the vessel wall.

MMPs belong to a family of zinc dependent endopeptidases and are mainly extracellular proteins, even though some members are also found intracellularly and may act on intracellular proteins. A typical MMP consists of a propeptide of about 80 amino acids, a catalytic metalloproteinase domain of about 170 amino acids, a linker peptide of variable lengths (also called the hinge region) and a hemopexin (Hpx) domain of about 200 amino acids [80]. MMPs can be subdivided according to substrate specificity, sequential similarity and domain organization into: Collagenases (MMP-1, MMP-8, MMP-13), gelatinases (MMP-2, MMP-9), stromelysins, (MMP-3, MMP-10, MMP-11), metrilysins (MMP-7, MMP-26), membrane-type MMPs (MMP-14, MMP-15, MMP-16, MMP-17, MMP-25), and other MMPs (MMP-20, MMP-26) [81]. Several MMPs have been implicated in age related pathologies. Below, the most well-known relationships with vascular aging are presented (Table 1).

#### 2.3.1. MMP-2

MMP-2 is a gelatinase which degrades the basement membrane proteins collagen IV, fibronectin, and laminin as well as fibrillar collagen peptides. In human aortas, evidence of enhanced MMP-2 activity with aging has been observed [106]. From an experimental point of view, the activation of MMP-2 has been associated with increases in AngII signaling, proinflammation, fibrosis, and elastin fragmentation [82,107]. In this regard, it has been shown that MMP-2 stimulates the TGF-β1 and SMAD signaling which result in increased VSMC collagen production within the vascular wall, myofibroblasts’ activation, and increased infiltration by monocytes/macrophages, therefore leading to inflammation and vascular injury [70,82]. In addition to this, MMP-2 impairs endothelial function by decreasing NO production [83]. Moreover, MMP-2 activation by AngII infusion to young rats has been associated with increased intima-media thickness (IMT) and vascular fibrosis, similar to alterations observed in untreated old control rats [70]. On the other hand, it has been shown that MMP-2 inhibition markedly reduces elastin fiber degeneration, collagen deposition, and blood pressure (BP) increase [84]. Finally, more recently, it was demonstrated that therapeutic knockdown of MMP-2 significantly attenuates the age-dependent carotid stiffness by mechanisms of blunted elastin fragmentation and enhanced eNOS activation [85].

#### 2.3.2. MMP-3

MMP-3 enzyme is known to degrade collagen types II, III, IV, IX and X, proteoglycans, fibronectin, laminin, and elastin. From a pathophysiological perspective, MMP-3 has a very important role in tissue remodeling, wound repair, and tumor initiation. So far, there is no evidence supporting increased MMP-3 production in healthy individuals except those of high cardiovascular risk [108]. Moreover, MMP-3 has been identified in atherosclerotic plaques, being expressed mainly by macrophages and activated VSMCs. Interestingly, MMP-3 has a dual role in atherosclerosis. Relative to this, it has been demonstrated in ApoE mice that the loss of MMP-3 is associated with extensive atherosclerotic plaque formation but reduced aneurysm formation [87]. A similar MMP-3 knockout study has also confirmed an accelerated plaque growth rate with increased macrophage and decreased VSMC composition, leading to the formation of unstable plaques [88]. In addition, MMP-3 activation by the factor Forkhead box O3a (FOXO3a), has been implicated as a mediator of vascular detachment and apoptosis of ECs [86].

#### 2.3.3. MMP-7

MMP-7 is a matrilysin mainly produced by the macrophages that has a low catalytic capacity for ECM structural proteins. However, MMP-7 has the widest portfolio of biologically active proteins/peptide substrates of any MMP. These substrates include matrixines such as osteopontin, thrombospondin, and TGF-β, all profibrotic molecules in nature [109]. Normally, MMP-7 levels are found increased in patients with cardiovascular risk such as those with carotid atherosclerosis.Relative to this, enhanced MMP-7 expression and activity have been demonstrated within the atherosclerotic plaques linking MMP-7 to plaque instability by several mechanisms. In particular, it has been shown that within the atherosclerotic lesions, MMP-7 is primarily located to macrophages and predominantly in areas with less organized collagen fibers, therefore implying a potential influence of MMP-7 on collagen structure and plaque stability [89]. In addition to this, MMP-7 levels have been also associated with heightened atherosclerotic burden through cleavage of apolipoprotein A-IV and the establishment of an oxidative environment [90]. Moreover, it has been demonstrated that MMP-7 mediates cleavage of n-cadherin, which is a cell-cell junction protein, and thus promotes VSMC apoptosis [91]. At the same time, deletion of MMP-7 in ApoE knockout mice leads to a significant increase in VSMC content within the plaques, thus contributing to higher instability [88]. Finally, concerning other vascular properties, MMP-7 has been implicated in the modulation of vascular tone, through arterial shedding of the heparin-binding epidermal growth factor (HB-EGF) and subsequent activation of the EGF receptor leading to vasoconstriction [92].

#### 2.3.4. MMP-9

MMP-9 is a gelatinase with a very low catalytic capacity for structural proteins that is involved in the proteolytic activation of other important biologically active molecules such as TGF-β, and other “pro-fibrotic” proteins [110]. So far, evidence regarding association of MMP-9 levels with healthy aging is lacking, howerer, MMP-9 has been found to be consistently increased in patients with cardiovascular disease [111] and has been mainly involved in the inflammatory process of atherosclerosis. As such, it has been experimentally demonstrated that MMP-9 is able to induce certain pro-inflammatory and pro-apoptotic activities in the ECs mainly through cleavage of the protease activated receptor-1 (PAR-1) [93]. Furthermore, MMP-9 has been found to promote migration of VSMCs and contribute to plaque destabilization in human carotid atherosclerotic plaques via increased vascular endothelial growth factor (VEGF) production and neovascularization [94,112]. On the other hand, MMP-9 knockout in ApoE mice has been shown to reduce the size of atherosclerotic lesions as well as plaque burden [95,96]. Similarly, loss of MMP-9 function has been consistently shown to inhibit VSMC migration, restrict vascular remodeling and prevent dilatation of the aorta and, hence, the formation of aneurysms [97,98].

The contribution of MMPs in vascular aging has been further corroborated by the observations of vascular impact upon MMP inhibition. It has been shown that tissue inhibitors of MMPs (TIMPs) including four molecules (TIMP-1, -2, -3, -4), reversibly inhibit the proteolytic activity of functional MMPs and an imbalance of MMPs and TIMPs has been implicated in hypertension, atherosclerotic plaque formation and aortic aneurysm formation in several experimental models [113]. More specifically, it has been demonstrated that overexpression of TIMP-1 by gene transfer can reduce balloon injury-induced intimal formation while TIMP-3 deficiency enhances inflammation and aggravates atherosclerosis in ApoE-knockout mice [104]. In addition to this, TIMP-3 has been demonstrated to mediate the inhibitory effect of interleukin-32α on endothelial inflammation, smooth muscle cell activation, and development of atherosclerosis [103]. Similar effects have been demonstrated for TIMP-2, and TIMP-4, mainly through mechanisms of VSMC migration and apoptosis [101,114]. Furthermore, TIMP-1 appears to protect against aortic aneurysm formation and rupture in rat models since its overexpression prevents elastin degradation. Similarly, in response to AngII, TIMP-3 gene deletion in non-atherosclerotic mice has been shown to trigger adverse remodeling of the abdominal aorta evidenced by reduced aortic wall thickness due to loss of elastic lamellae and inflammation, thus suggesting that reversing TIMP-3 levels may confer protection against aneurysm progression and rupture [102].

To sum up, a wealth of data confirms that vascular aging is characterized by increased MMPs’ activity which has been firmly associated with endothelial inflammation (such as endothelial cell senescence/apoptosis/necrosis, thrombosis, and dysfunction), elastin fragmentation, fibrosis, calcification and atherogenesis. Therefore, a chronic increase in MMP activation is central to the aging-associated vascular alterations. On the other hand, MMPs inhibitors such as TIMPs, provide a rather protective mechanism to prevent excessive degradation of ECM and the consequent harmful effects of MMPs on vascular integrity. Therefore, a delicate balance exists between overexpression of MMPs and MMPs deficiency due to TIMPs, which in the case of vascular aging, tends towards MMPs overactivity. This imbalance is a dynamic process that can be altered in various vascular diseases (i.e., hypertension, atherosclerosis), however, generally an increased MMPs activity prevails. In any case, defining the certain contribution of MMPs and TIMPs in vascular aging is rather a very complex task, taking into account that an increase in one MMP in a certain vascular region may be paralleled by a decrease of other MMPs in other regions. In addition, due to certain differences in the proteolytic activities of MMPs towards different substrates, MMP activity may vary during the course of a disease.

### 2.4. Epigenetic Regulation

#### 2.4.1. DNA Methylation

DNA methylation is a dynamically reversible process that modifies the genome function through the addition of methyl groups to cytosine in order to form 5-methyl-cytosine (5mC) and it is regulated by DNA methyltransferases (DNMT1, DNMT3A and DNMT3B) and demethyltransferases. In general, DNA methylation and hypermethylation inhibit gene expression either by recruiting proteins which are implicated in gene repression or by impeding the binding of transcription factors to DNA [115]. On the other hand, DNA demethylation or hypomethylation preserves gene expression, although at a cost, since it can initiate transcription at an incorrect gene region or even exhibit high transcriptional activity in normally silent sites. Therefore, hypomethylation may cause structural changes, chromosome instability and expression of potentially harmful genes [116]. Accumulating evidence has identified several genes which are regulated through different levels of DNA methylation and are involved in the development of vascular aging by modulating the function of several vascular cells such as ECs, VSMCs and macrophages [117]. 

In this regard, it has been shown that under the influence of LDL, DNMT1 hypermethylates the endothelial Kruppel-like Factor 2 (KLF2) promoter region, therefore repressing its expression and causing EC inflammation and thrombosis [118]. In addition, it has been demonstrated that upregulation of DNMT1 by oscillatory shear stress increases ROS production, stimulates THP-1 monocyte adhesion in ECs and enhances expression of proliferating cell nuclear antigen (PCNA), ICAM-1 and VCAM-1, all mechanisms leading to accelerated EC migration, proliferation, and inflammation [119]. Similarly to ECs, DNA methylation regulates several functions of VSMCs including proliferation which is an essential mechanism of vascular damage. As such, it has been identified that hypermethylation of the Mitofusin-2 and Phosphatase and tensin homologue on chromosome 10 inhibits their pertinent transcription leading to VSMCs proliferation during atherosclerotic plaque formation [120]. In fact, differential DNA methylation levels have been recognized to play an important role in the initiation and propagation of atherosclerosis. Consistent with this, it has been demonstrated that elevated methylation levels of the forkhead box P3-Treg-specific demethylated region (FOXP3-TSDR) gene accelerate atherosclerosis by reducing the percentages of regulatory T cells [121]. Furthermore, promoter methylation changes occurring at AIRE1 and ALOX12 genes have been implicated as potential epigenetic alterations in the etiology of atherosclerotic plaques [122]. Importantly, large genome-wide analyses have revealed the predominance of extensive hypomethylation in atherosclerotic plaques which correlates with the expression of several genes implicated in atherogenesis such as RTL1, CDKN2B, and PLA2G7 [123,124]. Finally, from a clinical perspective, differentially methylated levels of BRCA1 and CRISP2 regions have been associated with subclinical atherosclerosis measures such as the coronary calcium score and carotid IMT in individuals with subclinical cardiovascular disease [125].

#### 2.4.2. Histone Modification

Histone modification is a process during which chromatin structure and function as well as gene expression, transcription and repair are regulated. Similarly, post translational modifications are also determined by this mechanism [126]. This regulation is enabled by the interaction between histone proteins and DNA. The mechanisms in charge of histone modification include acetylation, methylation, phosphorylation and ubiquitation. Accordingly, the main enzymes involved are histone acetyl transferases (HATs), deacetylases (HDACs), methyltransferases (HMTs) and demethylases (HDMs) [127].

HDACs are divided into 4 classes: Class I (HDACs 1,2,3,8), Class IIa (HDACs 4,5,7), Class IIb (HDACs 6 and 10), Class III (NAD dependent sirtuin [Sirt] enzymes [Sirt 1–7]) and Class IV (HDAC 11). Activity of HDACS is regulated by metabolic intermediates, such as NAD, Acetyl-CoA and beta-OH-butyrate [128]. Among all HDACs, sirtuins are the most widely studied and Sirt1 is the best characterized member in relation to vascular aging. 

Sirt1 expression in human arteries of young and old donors shows an inverse correlation with age [129]. In addition, Sirt1 is systematically expressed at vascular level by several cells including ECs, monocytes/macrophages and VSMCs and is implicated in deacetylation of several transcriptional factors, co-regulatory proteins and enzymes like peroxisome proliferator-activated receptor-γ coactivator-1α (PGC-1a), NF-κB, eNOS, FOXO, p53, p300/CBP, H3H9 and H3K56 [130]. Overall, previous studies have revealed in detail the protective role of Sirt1 against vascular aging through abundant beneficial effects in the structural and functional homeostasis of the vasculature [131]. More specifically, it has been demonstrated that endothelial Sirt1 stimulates NO production through increased eNOS activity which results by deacetylation of eNOS at lysine (Lys)-496 and Lys-506. On the contrary, Sirt1 knockdown results in decreased endogenous NO production and impaired endothelial-dependent vasodilatation [132]. Similarly, Sirt1 expression is reduced in ECs obtained from human arteries of older compared to younger adults, thus linking Sirt1 with endothelial dysfunction and the aging phenotype [133]. In close association, it has been observed that Sirt1 overexpression protects from stress-induced premature endothelial senescence through deacetylation of Lys-373, Lys-382, and Lys-320 [134]. More recently, Sirt1 downregulation was linked to increased nuclear accumulation of acetylated serine/threonine liver kinase B1 (LKB1) and hence irreversible structural alterations of the vascular wall including adverse arterial remodeling and vascular stiffness [135]. Central to its protective role against vascular aging, it has been also shown that Sirt1 possesses significant anti-oxidative, anti-inflammatory, and anti-atherosclerotic properties. As such, it has been demonstrated that Sirt1 regulates cellular oxidative stress via deacetylation and activation of the FOXO- 1, 3, and 4 transcription factors and the induction of multiple anti-oxidative enzymes [136]. Furthermore, Sirt1 deacetylates the NF-κB p65 subunit, therefore inhibiting the expression of several inflammation-related genes as well as pro-inflammatory cytokines [131]. Consequently, various preclinical and clinical settings have shown that certain Sirt1 pharmacological modulators display significant anti-inflammatory, anti-atherosclerotic and anti-oxidative properties [137,138]. A detailed summary of the mechanisms through which Sirt1 protects against vascular aging is depicted in Table 2.

Apart from Sirt1, Sirt6 has also demonstrated a protective function against vascular aging. Sirt6 has been well characterized as a highly specific H3 deacetylase that targets Lys-9 (H3K9), Lys-56 (H3K56), and Lys-18 (H3K18) and specifically represses the activities of several transcription factors involved in aging including NF-κB, c-JUN, and hypoxia-inducible factor 1-alpha (HIF-1α) [146]. Through these functions, Sirt6 has evolved as a key regulator in chromatin signaling, maintenance of telomere integrity, and prevention of genomic instability. At vascular level, Sirt6 is highly expressed in ECs and protects them against premature senescence. More specifically, it has been demonstrated that Sirt6 deletion by siRNAs inhibits ECs replication and promotes EC senescence. Consistent with this observation, Sirt6 deletion leads to increased mRNA expression of PAI-1 and ICAM-1 as well as reduced eNOS expression and, finally, a diminished ability of the ECs to form vessels in vitro [147]. Moreover, Sirt6 deletion has been closely associated with decreased expression of FOXM1, a critical transcription factor for cell cycle progression, therefore promoting endothelial senescence [148]. Interestingly, Sirt6 provides a significant anti-atherosclerotic effect by reducing the expression of multiple atherosclerosis-related genes, including the proatherogenic gene tumor necrosis factor superfamily member 4 (TNFS4). Furthermore, it promotes macrophage autophagy and inhibits the expression of VCAM-1, ICAM-1, and P-selectin. Collectively these functions lead to reduced infiltration of macrophages and foam cells and increased plaque stability [149,150]. 

In contrast to the well-documented protective actions of Sirt1 and Sirt6, the vascular functions of other Sirts and their association with vascular aging remain a matter of investigation with most evidence pointing towards the role of Sirt3. Sirt3 is considered to play a key role in metabolic regulation and ROS homeostasis via deacetylation of numerous mitochondrial enzymes. In this way, it has been demonstrated that Sirt3 deletion in AngII-treated ECs, significantly enhances ROS production and decreases eNOS activity and NO [151]. Accordingly, Sirt3 deletion has been shown to confer a highly deleterious impact on vascular homeostasis by inactivating SOD2, thus leading to mitochondrial oxidative stress and an inflammatory vascular phenotype defined by impaired vasorelaxation, and hypertrophy. On the contrary, increased Sirt3 expression prevented all the aforementioned deleterious effects [152].

On the other hand, scarce evidence regarding the role of Sirt2 exists. In an experimental study, Sirt2 knockdown in human umbilical ECs (HUVECs) under oxidative stress resulted in significant alterations in the expression of various genes implicated in several cellular protein and metabolic processes. Furthermore, pharmacologic Sirt2 inhibition attenuated the oxidative stress-induced EC death, hence implying that Sirt2 could be functionally important in ECs under stress [153]. Likewise, Sirt4 and Sirt 5 possess a minor role in vascular homeostasis and aging according to current information. Overexpression of Sirt4 has been found to mitigate the nuclear translocation and transcriptional activity of NF-κB thereby attenuating the endothelial expression of several pro-inflammatory factors including IL-1β, IL-6 and IL-8, COX-2, MMP-9, and ICAM-1 (Tao et al., 2015). Finally, silencing of Sirt7 in ECs has shown to compromise endothelial function by modulating TGF-β signaling [154].

#### 2.4.3. Non-Coding RNAs

The non-coding RNAs (ncRNAs) represent RNA molecules that lack protein coding potential and are divided, according to their nucleotide content, into short or small ncRNAs (<200 nucleotides) and long ncRNAs (>200 nucleotides). Furthermore, microRNAs (miRNAs) (21–25 nucleotides) belong to the short ncRNAs and are the most extensively studied member while recently discovered circular RNAs (300–500 nucleotides) pertain to the long ncRNAs. The ncRNAs play a significant role in the post-transcriptional genetic regulation. In particular, miRNAs negatively regulate gene expression by binding a target mRNA and inducing its degradation or by inhibiting its translation [155]. 

Increasing evidence has shown that miRNAs have a considerable impact on various molecular mechanisms related to vascular function and aging (Table 3). More specifically, miRNAs are differentially implicated in the epigenetic regulation of vascular senescence, oxidative stress, inflammation, and calcification [156]. In this regard, it has been demonstrated in proatherogenic apoE−/− mice that increased levels of miR-217 downregulate Sirt1 expression and crucial eNOS activators, including the apelin receptor and VEGF pathways, thus resulting in reduced NO production and endothelial dysfunction. This observation was associated with a harmful impact on vascular homeostasis due to accelerated atherosclerosis, increased BP, and cardiac dysfunction. On the other hand, it has been shown that miR-217 inhibition delays cellular senescence, reduces the development of atherosclerosis and improves vascular function [156,157]. Importantly, miR-217 has been found to be among the most highly induced miRNAs in aging ECs and has been associated with well-established cardiovascular risk factors in human cohorts highlighting its role as a biomarker of human aging [158]. Correspondingly, miR-10A and miR-21 have been implicated in endothelial progenitor cell senescence through suppression of the high-mobility group A2 molecule while miR-34a acts by suppressing Sirt1 [159,160]. In addition, several miRs can differentially regulate the phenotypic transition of the VSMCs during vascular aging. More specifically, it has been shown that miR-146a targets the KLF4 3’-untranslated region, further promoting VSMC proliferation in vitro and vascular neointimal hyperplasia in vivo [161]. On the contrary, miR-143 and miR-145 synergistically target a network of transcription factors, including myocardin, and Elk-1 in order to promote normal differentiation and repress proliferation of VSMCs, and their levels have been found to be downregulated in injured or atherosclerotic vessels [162]. A similar anti-proliferative role has been ascribed to overexpression of miR-128 and miR-22, both in vitro and in vivo, in injured mouse carotid and human femoral arteries [163,164]. Finally, several miRs including miR-126, miR-146a and miR-155 have been implicated in inflammaging through various mechanisms such as NF-κB and toll-like receptor (TLR) signaling, increased expression of cell adhesion proteins (VCAM-1) and activation of inflammatory cells [165,166].

### 2.5. Telomere Shortening

Telomeres are non-coding DNA structures consisting of a repetitive hexanucleotide DNA sequence (TTAGGG) found in the terminal loops, where they cap and stabilize the physical ends of eukaryotic chromosomes [168]. While aging telomeres shorten with each successive cell division, however, below a critical length, they induce the DNA damage response (DDR), eventually leading to replicative senescence and the end of cellular proliferation. Actually, telomere shortening or attrition constitutes a major triggering factor of senescence leading to vascular aging and cardiovascular disease [169,170]. 

Abundant experimental data have linked telomere shortening with the development of endothelial dysfunction and atherogenesis [171]. More specifically, studies in mice have shown that critically short telomeres resulting from telomerase deficiency induce endothelial dysfunction in vascular tissue [172] whereas the introduction of the telomeric repeat-binding factor 2 (TRF-2), a protective component of telomerase, in human ECs extends the cellular lifespan and ameliorates endothelial-dependent vasodilation [173]. In addition, reduced telomere length has been related to the presence of atherosclerosis. Notably, markedly shorter telomers have been identified in ECs and VSMCs in human atherosclerotic plaques compared to non-atherosclerotic lesions, where they have been correlated with atherosclerotic plaque severity and accelerated atherogenesis [171,174,175]. Conversely, longer telomers have been found in vascular segments resistant to atherosclerosis [176]. 

Furthermore, clinical data have highlighted the association of telomere length with arterial stiffness and atherosclerotic burden across different age and cardiovascular risk populations but also healthy individuals; hence, shorter telomeres have been associated with increased aortic pulse wave velocity [177,178], pulse pressure [179] and carotid IMT [180]. In addition, leukocyte telomere length is decreased in patients with various cardiovascular disease phenotypes including heart failure, myocardial infarction [181,182] and atherosclerotic hypertensive disease [183]. More importantly, telomere length has been closely correlated with cardiovascular risk in several large cohorts and meta-analyses. In particular, shortened leukocyte telomeres have been associated with a higher risk of coronary heart disease including myocardial infarction independently of conventional cardiovascular risk factors [184,185] as well as a higher risk of all-cause mortality [186].

### 2.6. Cellular Senescence

Cellular senescence is a state of a durable, irreversible cell-cycle arrest of previously replication-competent cells [187] which plays a dual role in physiology and disease [188]. In this regard, transient induction of senescence followed by tissue remodeling has been recognized as a beneficial mechanism to eliminate damaged or aged cells. Conversely, persistent senescence and inability to eliminate the excess damaged cells has been linked with detrimental effects [189]. 

Senescence has been recognized as a central hallmark of aging since most of its stimuli including telomere attrition, mitochondrial dysfunction, oncogene activation, and DNA damage, are primary drivers of the process. Importantly, senescence is also by itself a key driver of vascular dysfunction and aging by mediating endothelial dysfunction, inflammation, and atherosclerosis [188,190,191]. More specifically, early in vitro observations have shown that induction of senescence in human aortic ECs reduces levels of NO and increases expression of ICAM-1 [173]. Moreover, ex vivo observations have shown that aorta rings of mice expressing high levels of the senescence-selective markers cyclin-dependent kinase inhibitors (p16INK4a and p19ARF) present impaired endothelium-dependent vasodilation [172].

Τhe senescence-associated secretory phenotype (SASP) consists of a plethora of factors produced by the senescent cells including pro-inflammatory cytokines and chemokines, growth modulators, angiogenic factors, and MMPs that can induce inflammation, stem cell dysfunction, immunity activation, apoptosis and further trigger senescence in neighboring cells [189,192]. The net result is a state of persistent chronic inflammation, known as inflammaging which is tightly associated with multiple age-related phenotypes [37,193]. In close association with this, experimental data have documented considerable accumulation of senescent VSMCs and ECs in human atherosclerotic lesions that persistently express key SASP factors [194] and lead to a highly inflammatory and pro-atherogenic environment which contributes to the progression of atherosclerosis [195,196]. Moreover, it has been shown that calcium enriched microvesicles produced by senescent human ECs promote vascular calcification, a surrogate marker of atherosclerosis and aged vascular disease [197]. Furthermore, studies in humans have demonstrated that increased levels of SASP circulating proteins are associated with clinically apparent aging phenotypes such as frailty [37]. Indeed, various circulating SASP components significantly increase with chronological or advanced biological aging, as measured by the frailty index. In addition, several SASP proteins exhibit high sensitivity and specificity for adverse outcomes risk prediction in certain aged individuals [198]. 

Additionally, strong evidence advocating the contribution of senescence to vascular aging comes from preclinical studies investigating pharmacologic agents which lead to the “senolytic” clearance of senescent cells and attenuation of inflammation [189,199]. Pertinent to this, administration of the senolytic drugs Dasatinib and Quercetin in chronologically aged mice leads to a significant reduction in the number of senescent cells and improved vasomotor function [200]. In addition, it has been shown that Navitoclax, another senolytic agent, hinders the progression of atherosclerosis in transgenic models of LDL receptor-deficient mice by selectively removing senescent macrophages from atherosclerotic plaques [195]. Finally, alternative pharmacologic approaches have emerged including drugs that prevent the progression of cell senescence without inducing the death of senescent cells (senomorphic drugs) such as SASP inhibitors. To this end, Rapamycin (Sirolimus) favors the clearing of dysfunctional senescent cells, ameliorates endothelial function and improves large artery stiffness [201]. Likewise, Resveratrol prevents the increase in pulse wave velocity and decreases vascular inflammation observed in non-human primates exposed in high fat/sucrose diet [202].

### 2.7. Autophagy

Autophagy is a highly selective physiological process by which cells encapsulate and deliver their macromolecular components such as proteins and organelles to lysosomes for subsequent degradation [203]. An increasing amount of evidence has highlighted the critical role of autophagy as being an essential mechanism to both preserve cellular homeostasis (through the removal of wasteful cellular products) and provide a survival adaptive mechanism for cells during stressful metabolic demands [204]. Importantly, with aging, there is a progressive reduction in the autophagic activity across several species and model systems [205,206], which has been further associated with vascular dysfunction, accelerated aging, and several age-related vascular diseases [207]. 

More specifically, within the vasculature, impaired autophagy has been closely linked to the establishment of oxidative-induced senescence and the propagation of a highly inflammatory microenvironment [208,209]. Moreover, data from aged mice and human subjects have shown that compromised autophagy of ECs is associated with a markedly blunted endothelial-dependent vasodilative response [210]. Coincident with this effect, it has been demonstrated that loss of autophagy promotes an increase in endothelial ROS and inflammatory cytokines, hence suggesting that autophagy may regulate vascular homeostasis, in part, through a NO-dependent pathway [211]. Furthermore, defective autophagy has been implicated in angiogenesis, calcification of the vessel wall and atherosclerosis [11,212].

Contrary to the harmful effects of impaired autophagy, several lines of evidence have corroborated that induction of autophagy has a protective effect on vascular homeostasis. In this context, genetic manipulations in multiple short-lived model organisms have indicated that activation of autophagy significantly extends organismal lifespan, thus pointing out the role of autophagy as a tool to promote longevity [213]. In addition, the lifestyle modification of caloric restriction, which is the most effective strategy to induce autophagy so far, has been shown to improve vascular function in both rodent models and human subjects by intervening in crucial regulatory pathways including the deacetylase Sirt1, the AMP-activated protein kinase (AMPK), and the mammalian target of rapamycin (mTOR) [204,205]. In fact, caloric restriction has been shown to be one of the most powerful lifestyle-based strategies for extending maximal lifespan and health span in rodents. Regarding vascular aging, it has been shown that long-term caloric restriction in mice prevents the age-related declines in endothelial function and increases in large elastic artery stiffness and these effects are related to reduced oxidative stress [214]. Likewise, short-term (i.e., 3–8 weeks) caloric restriction also reverses the age-related vascular dysfunction in old mice. Additionally, in humans, caloric restriction-based weight loss in overweight and obese middle-aged and older adults has been shown to improve macrovascular and microvascular endothelial function and large elastic artery stiffness [215]. Finally, pharmacological interventions such as spermidine and trehalose have shown to improve vascular aging by ameliorating endothelial dependent function and arterial stiffening [210]. 

## 3. Conclusions

Vascular aging and the associated changes in the vascular wall represent a certain hallmark of the aging process that are irrefragably related to increased cardiovascular mortality and the development of several age-related pathologies. Accumulating evidence over the last years has called attention on the several complex molecular pathways implicated in the pathophysiology of vascular aging which are a matter of intense investigation. Among them, oxidative stress, atherosclerosis, vascular inflammation and the related endothelial dysfunction, seem to represent the common denominator that accelerates vascular ageing and stiffening of the arteries. Within this conceptual framework, a deeper understanding of these highly sophisticated biological processes is warranted in order to develop certain therapeutic targets and facilitate future interventions aiming to improve human health span and longevity.

## Figures and Tables

**Table 1:** Major matrix metalloproteinases along with their relative inhibitors and their involvement in vascular aging.

MMP and TIMP Class	*Overexpression*	*Deficiency*
**MMP-2**	(a) Increased TGF-β1 and SMAD signaling leading to - VSMC collagen production - myofibrolast activation - infiltration by monocytes/macrophages and inflammation [70,82] (b) Endothelial dysfunction due to decreased NO production [83] (c) Increased intima-media thickening and vascular fibrosis [70]	(a) Reduced elastin fiber degeneration and collagen deposition [84] (b) Enhanced eNOS activation [85]
**MMP-3**	Apoptosis of ECs [86]	Accelerated plaque growth rate with increased macrophage and decreased VSMC composition [87,88]
**MMP-7**	(a) Atherosclerosis and plaque instability through collagen and matrix modulation and cleavage of apolipoprotein A-IV [89,90] (b) VSMC apoptosis through cleavage of n-cadherin [91] (c) Vasoconstriction through shedding of the HB-EGF and subsequent activation of EGFR [92]	Increased accumulation of VSMCs within the atherosclerotic plaques
**MMP-9**	(a) Apoptosis in ECs through cleavage of PAR-1 [93] (b) Migration of VSMCs and contribution to atherosclerotic plaque instability and intraplaque hemorrhage [94]	(a) Reduction in size of atherosclerotic lesions and plaque burden [95,96] (b) Inhibition of VSMCs’ migration and restriction of vascular remodeling [97] (c) Prevention of formation of abdominal aortic aneurysms [98]
**TIMP-1**	(a) Reduction in intimal formation through decreased collagen deposition and increased elastin accumulation [99] (b) Protection against aneurysm formation and rupture through prevention of elastin degradation [100]	
**TIMP-2**	Suppression of atherosclerotic plaque progression through inhibition of migration and apoptosis of macrophages and foam cells [101]	
**TIMP-3**	(a) Reduced intimal formation through apoptosis of VSMC [102] (b) Atherosclerosis through inhibition of EC inflammation and VSMC proliferation and migration [103] (c) Accumulation of inflammatory monocytes/macrophages within the vascular wall [104]	(a) Enhanced inflammation and atherosclerosis through increased (b) Adverse vascular remodeling and vascular aneurysm formation through loss of elastic lamellae and inflammation [105]

EC: endothelial cell; EGFR: epidermal growth factor receptor; eNOS: endothelial nitric oxide synthase; HB-EGF: heparin-binding epidermal growth factor; MMP: matrix metalloproteinase; NO: nitric oxide; PAR-1: protease activated receptor-1; TGF-β1: tissue growth factor-β1; VSMC: vascular smooth muscle cell.

**Table 2:** Beneficial mechanisms through which SIRT1 upregulation protects against vascular aging.

Recruitment of EC migration [139]
Delay of the aging and dysfunction of EPCs [140]
Inhibition of aging of ECs by binding the PAI-1 promoter and by deacetylation of histone H4K16 [141]
Promotion of endothelial KLF2 expression which enables transition of ECs to a “vaso-protective” state [141]
Mitigation of hyperglycaemia-induced endothelial dysfunction due to ROS production by inhibiting vascular *p66Shc* gene transcription [142]
Alleviation of oxidative stress and inflammation by the inhibition of NF-κB signaling pathway [143]
Activation of eNOS and promotion of NO production by the deacetylation of eNOS on Lys496 and Lys506 [132]
Reduction of COX-2 expression through downregulation of transcription factor AP-1 in macrophages [144]
Reduction of arterial remodeling and stiffness by alleviation of oxidative stress in VSMCs [145]
Deacetylation and activation of the FOXO 1, 3, and 4 transcription factors leading to the expression of several antioxidant genes [136]

AP-1: activator protein-1; COX-2: cyclooxygenase-2; ECs: endothelial cells; eNOS: endothelial nitric oxide synthase; EPCs: endothelial progenitor cells; FOXO: forkhead fox; HUVECs: human umbilical vein endothelial cells; KLF2: Kruppel-like factor 2; NF-κB: nuclear factor kappa B; NO: nitric oxide; PAI-1: plasminogen activator inhibitor-1; PARP: Poly (ADP-ribose) polymerase; ROS: reactive oxygen species; VSMCs: vascular smooth muscle cells.

**Table 3:** Major miRNAs and their involvement in vascular aging.

miR-10A	Propagation of senescence of EPCs through suppression of the high-mobility group A2 molecule [160]
miR-21	Propagation of senescence of EPCs through suppression of the high-mobility group A2 molecule [160]
miR-22	Inhibition of VSMC proliferation and migration and neointima formation 164
miR-34a	Suppression of EC proliferation and promotion of EC senescence in part through Sirt1 inhibition [159] Impairment of EPC-mediated angiogenesis through suppression of silent information regulator 1 [159]
miR-126	Reduction of endothelial inflammation through inhibition of VCAM-1 expression [165]
miR-128	Reduction of VSMC proliferation, migration, and contractility [163]
miR-143	Inhibition of VSMC proliferation through targeting the transcription factor Elk-1 [162]
miR-145	Inhibition of VSMC proliferation through targeting the transcription factor myocardin [162]
miR-146a	Promotion of VSMC proliferation and vascular neointimal hyperplasia through targeting KLF4 [161]
miR-155	Promotion of atherosclerosis through repression of macrophage BCL6 expression [167] Endothelial dysfunction and vasoconstriction through downregulation of eNOS and sGCβ1 expression [166]
miR-217	Acceleration of EC senescence, endothelial dysfunction and development of atherosclerosis through Sirt1 downregulation [157,158]

BCL6: B-cell lymphoma 6 protein; BP: blood pressure; EC: endothelial cell; EPC: endothelial progenitor cell; eNOS: endothelial nitric oxide synthase; KLF4: Krüppel-like factor 4; sGCβ1: soluble guanylyl cyclase β1; VCAM-1: vascular cell adhesion molecule-1; VEGF: vascular endothelial growth factor; VSMC: vascular smooth muscle cells.

## Supplementary Materials

The supporting information can be downloaded at: https://www.mdpi.com/article/10.3390/ijms23158672/s1.

## Abbreviations

ACE	angiotensin converting enzyme
AMPK	AMP-activated protein kinase
AngII	angiotensin II
AP-1	activator protein-1
ApoE	apolipoprotein E
BCL6	B-cell lymphoma 6 protein
BP	blood pressure
CAT	catalase
COX-2	cyclooxygenase-2
CRP	C-reactive protein
CTGF	connective tissue growth factor
CVD	cardiovascular disease
DDR	DNA damage response
DNMT	DNA methyltransferases
ECM	extracellular matrix
ECs	endothelial cells
EGFR	epidermal growth factor receptor
EPCs	endothelial progenitor cells
eNOS	endothelial nitric oxide synthase
FOXO	Forkhead box
GPX	glutathione-peroxidase
HATs	histone acetyltransferases
HB-EGF	heparin-binding epidermal growth factor
HDACs	histone deacetylases
HDMs	histone demethylases
HDL	high density lipoprotein
HIF-1α	hypoxia-inducible factor 1-alpha
HMTs	histone methyltransferases
Hpx	hemopexin
HUVECs	human umbilical endothelial cells
ICAM-1	intercellular adhesion molecule-1
IFN	interferon
IL	interleukin
IMT	intima-media thickness
KLF2	Kruppel-like Factor 2
LDL	low density lipoprotein
LKB1	liver kinase B1
LOX-1	lectin-like oxidized low-density lipoprotein receptor-1
LPOs	lipoxygenases
Lys	lysine
MAPKs	mitogen-activated protein kinases
MCP-1	monocyte chemotactic protein-1
MIP-1a	macrophage inflammatory protein-1 alpha
miRNAs	microRNAs
MMPs	matrix metalloproteinases
MPOs	myeloperoxidases
mTOR	mammalian target of rapamycin
NADPH	nicotinamide adenine dinucleotide phosphate
ncRNAs	non-coding RNAs
NF-κB	nuclear factor-kb
NO	nitric oxide
NOS	nitric oxide synthase
oxLDL	oxidized low-density lipoprotein
PAI-1	plasminogen activator inhibitor-type 1
PAR-1	protease activated receptor-1
PARP	poly (ADP-ribose) polymerase
PDGF	platelet-derived growth factor
PCNA	proliferating cell nuclear antigen
PGC-1a	peroxisome proliferator-activated receptor-γ coactivator-1α
RAS	renin/angiotensin system
RONS	reactive oxygen and nitrogen species
ROS	reactive oxygen species
RSPO3	pro-permeability factor R-spondin 3
SASP	senescence-associated secretory phenotype
sGCβ1	soluble guanylyl cyclase β1
Sirt	sirtuin
SOD	superoxide dismutase
TGF-β	transforming growth factor-β
TIMPs	tissue inhibitors of matrix metalloproteinases
TLR	toll-like receptor
TNF	tumor necrosis factor
TNFS4	tumor necrosis factor superfamily member 4
TRF-2	telomeric repeat-binding factor 2
VCAM-1	vascular cell adhesion molecule-1
VEGF	vascular endothelial growth factor
VSMC	vascular smooth muscle cell
