# Supplementary material for: Theories and Molecular Basis of Vascular Aging: A Review of the Literature from VascAgeNet Group on Pathophysiological Mechanisms of Vascular Aging
Source: Int J Mol Sci. 2022 Aug 4;23(15):8672. doi: 10.3390/ijms23158672 (PMC9368987; doi:10.3390/ijms23158672)
Supplement: Supplementary file 1 [file ijms-23-08672-s001.zip › ijms-1810517-supplementary.pdf]

International Journal of *Molecular Sciences*

MDPI

Review: Supplement

Theories and molecular basis of vascular aging: a review of the literature from VascAgeNet group on pathophysiological mechanisms of vascular aging

Eugenia Gkaliagkousi [1,*], Antonios Lazaridis [1], Soner Dogan [2], Emil Fraenkel [3], Bilge Guvenc Tuna [4], Ioana Mozos [5], Milica Vukicevic [6], Ozlem Yalcin [7] and Kristina Gopcevic [8,*]

[1] 3rd Department of Internal Medicine, Papageorgiou Hospital, Faculty of Medicine, Aristotle University of Thessaloniki, Greece; spanbiol@hotmail.com
[2] Department of Medical Biology, School of Medicine, Yeditepe University, Istanbul, Turkey; dogansoner@yahoo.com
[3] 1st Department of Internal Medicine, University Hospital and Pavol Jozef Šafárik University in Košice, Trieda SNP 1, 04066 Košice, Slovakia; emil.fraenkel@hotmail.com
[4] Department of Biophysics, School of Medicine, Yeditepe University, Istanbul, Turkey; bilgeguv@gamil.com
[5] Department of Functional Sciences-Pathophysiology, Center for Translational Research and Systems Medicine, "Victor Babes" University of Medicine and Pharmacy, 300173 Timisoara, Romania; ioanamozos@umft.ro
[6] Cardiac Surgery Clinic, Clinical Center of Serbia, Belgrade, Serbia; milicavukicevicmd@gmail.com
[7] Department of Physiology, School of Medicine, Koc University, Istanbul, Turkey; ozlemyalcin@ku.edu.tr
[8] Laboratory for Analytics of Biomolecules, Department of Chemistry in Medicine, Faculty of Medicine, Belgrade, Serbia
* Correspondence: egkaliagkousi@auth.gr (E.G.); kristinagopcevic@yahoo.com or kristina.gopcevic@med.bg.ac.rs (K.G.)

Table S1. Reactive oxygen and nitrogen species, radicals and nonradicals, their formation, characteristics and detoxification.

Species	Formula	Half life	Formation	Characteristics	Detoxification
Superoxide	$O_2^{\bullet-}$	10^{-6} s	$O_2 + e^- \rightarrow O_2^{\bullet-}$<br>$O_2 + Fe^{2+} \rightarrow Fe^{3+} + O_2^{\bullet-}$ (auto-oxidation)<br>$O_2^{\bullet-} + O_2^{\bullet-} + 2H_2O$ (Cu,Zn,Mn-SOD) $\rightarrow H_2O_2 + O_2$	Highly unstable, signaling function, synaptic plasticity	SOD (enzymatic), Vit C (non-enzymatic)
Hydrogen peroxide	H_2O_2	Stable	$O_2^{\bullet-} + O_2^{\bullet-} + 2H_2O$ (Cu,Zn,Mn-SOD) $\rightarrow H_2O_2 + O_2$	Cell toxicity, signaling function, generation of other ROS	CAT (enzymatic)
Hydroxyl radical	$OH^{\bullet}$	10^{-10} s	$Fe^{2+} + H_2O_2 \rightarrow Fe^{3+} + OH^{\bullet} + OH^-$ (Fenton reaction)<br>$O_2^{\bullet-} + H_2O_2 \rightarrow O_2 + OH^{\bullet} + OH^-$ (Haber-Weiss reaction)	Free radical, highly reactive, very reactive agent	Vit E (non-enzymatic)
Alkoxyl radical	$RO^{\bullet}$	10^{-6} s	$ROOH + Fe^{3+} \rightarrow RO^{\bullet} + Fe^{2+}$<br>$ROOH + HO_2^{\bullet} \rightarrow RO^{\bullet} + O_2$	Free radical, reaction product of lipids	Vit C
Peroxyl radical	$ROO^{\bullet}$	17 s	Protonation of $O_2^{\bullet-}$	Free radical, reaction product of lipids	β-carotene
Hypochlorite anion	OCl^-	Stable (min)	$H_2O_2 + Cl^-$ (MPO) $\rightarrow OCl^- + OH^{\bullet}$ from activated neutrophils at the site of inflammation	Reactive oxygen species, reactive chlorine species, enzymatically generated by myeloperoxidase	Vit C
Singlet oxygen	$^1O_2$	10^{-5} s	$HOCl + H_2O_2 \rightarrow {}^1O_2 + H_2O + Cl^-$	Induced/excited oxygen molecule, radical and non-radical form	β-carotene
Nitric oxide	$^{\bullet}NO$	s	L-Arg + O_2 + NADPH (NOS)$\rightarrow$L-Citrulline + $^{\bullet}NO$ + $NADP^+$	Environmental toxin, endogenous signal molecule	$O_2^{\bullet-}$
Peroxynitrite	$ONOO^-$	10^{-3} s	$O_2^{\bullet-} + {}^{\bullet}NO \rightarrow OONO^-$	Highly reactive reaction intermediate of $O_2^{\bullet-}$ and $^{\bullet}NO$	GP_X
Nitrogen dioxide	$NO_2^{\bullet}$	s	$HONOO \rightarrow NO_2^{\bullet}$	Highly reactive radical, environmental toxin	γ-Tocopherol
Nitrogen oxides	NO_x	s	$NO + O_2 + H_2O \rightarrow NO_3^- + NO_2^-$<br>$^{\bullet}NO + e \rightarrow NO_2^{\bullet+}$ nitrosonium cation<br>$^{\bullet}NO - e \rightarrow NO^-$ nitroxyl anion<br>$NO_2^{\bullet+} + NO^- \rightarrow N_2O + OH^{\bullet}$ etc.	Environmental toxins including NO and $NO_2^{\bullet}$ derived from combustion process	Some bacteria

CAT: catalase; GPx: glutathione peroxidase; NO: nitric oxide; SOD: Superoxide dismutase, Vit: vitamin.
